# Stabilization of G-Quadruplex-Duplex Hybrid Structures Induced by Minor Groove-Binding Drugs

**DOI:** 10.3390/life12040597

**Published:** 2022-04-18

**Authors:** Lily Scott, Tigran V. Chalikian

**Affiliations:** Department of Pharmaceutical Sciences, Leslie Dan Faculty of Pharmacy, University of Toronto, 144 College Street, Toronto, ON M5S 3M2, Canada; lilyf.scott@mail.utoronto.ca

**Keywords:** quadruplex-duplex hybrids, minor groove binding drugs, thermodynamics, stability, optical spectroscopy

## Abstract

Once it had been realized that G-quadruplexes exist in the cell and are involved in regulation of genomic processes, the quest for ligands recognizing these noncanonical structures was underway. Many organic compounds that tightly associate with G-quadruplexes have been identified. However, the specificity of G-quadruplex-binding ligands towards individual structures remains problematic, as the common recognition element of these ligands is the G-tetrad. In this paper, we focus on G-quadruplex-duplex hybrids (QDH) containing a hairpin duplex incorporated as a stem-loop into the G-quadruplex core. The presence of a stem-loop renders QDH amenable to sequence-specific recognition by duplex-binding drugs. Should the thermodynamic crosstalk between the stem-loop and the tetraplex core be sufficiently strong, the drug binding to the loop would lead to the stabilization of the entire structure. We studied the stabilizing influence of the minor groove-binders netropsin and Hoechst 33258 on a family of QDH structures, as well as a G-quadruplex and a hairpin modeling the G-quadruplex core and the stem-loop of the QDH’s. We found that the binding of either drug results in an enhancement of the thermal stability of all DNA structures, as expressed by increases in the melting temperature, T_M_. Analysis of the hierarchical order of increases in T_M_ revealed that the drug-induced stabilization arises from drug binding to the G-quadruplex domain of a QDH and the stem-loop, if the latter contains an all-AT binding site. This result attests to the thermodynamic crosstalk between the stem-loop and the tetraplex core of a QDH. Given the existing library of minor groove-binding drugs recognizing mixed A·T and G·C DNA sequences, our results point to an untapped avenue for sequence-specific recognition of QDH structures in vitro and, possibly, in vivo; thereby, opening the way for selective stabilization of four-stranded DNA structures at predetermined genomic loci, with implications for the control of genomic events.

## 1. Introduction

G-quadruplexes have entered the fields of biophysics and the structural biology of nucleic acids, as well as those of molecular and cell biology and nanotechnology, as previously unappreciated secondary structures that may act as regulatory elements in genomic events, as well as cation-responsive structural components in DNA-based nanotechnological devices [1,2,3,4,5,6,7,8,9,10]. Once the regulatory properties of G-quadruplexes were recognized, the quest for low molecular weight ligands (drugs) that can recognize and stabilize individual genomic G-quadruplexes has been underway [3,10,11,12,13,14,15]. These studies have identified a number of high-affinity ligands that bind to G-quadruplexes [13,14,15]. For all such drugs, the main recognition element is the G-tetrad, which is the common structural building block for any G-quadruplex. The ensuing challenge facing research groups working on the rational design of G-quadruplex-binding drugs is to discriminate between G-quadruplex structures. One strategy to meet this challenge is to equip the drug with functional groups that sample, in addition to G-tetrads, grooves and loops that would render the drug selective with respect to a particular G-quadruplex structure [14,16]. An alternative and potentially propitious approach is to concentrate on G-quadruplex-duplex hybrids (QDH), a sub-class of four-stranded DNA structures.

A QDH incorporates a stem-loop duplex linked to the G-quadruplex core via a duplex-quadruplex junction [17,18,19]. More than 80,000 DNA sequences capable of folding into QDH structures with stem-loops of 20 nucleotides or less have been identified across important regulatory sites in the human genome, including transcription/mutagenesis hotspots and cancer-associated genes [19]. The folding topologies of various QDH structures have been characterized in vitro by NMR [19,20,21,22]. The stem-loop in QDH may be positioned in a coaxial or orthogonal orientation relative to the G-quadruplex core, and the topology of the core is sensitive to the orientation of the loop [17]. QDH structures are thermodynamically stable and melt via two-state transitions, as revealed in the careful calorimetric and spectroscopic studies by Giancola and colleagues [23].

The presence of a double-stranded hairpin loop renders a QDH amenable to recognition by minor groove-binding drugs, which can be specifically targeted to the sequence of the loop [24]. Drug binding to double stranded DNA leads to an increase in the stability of the host duplex [25,26,27,28]. Depending on the extent of the cooperative link between the double-stranded stem-loop and the tetraplex core of QDH, the drug binding to the stem-loop may or may not cause a global increase in the stability of the hybrid structure. Should the cooperativity be sufficiently strong to allow the drug-induced stabilization of the loop to propagate to the tetraplex core, the sequence-specific association of a minor groove-binding drug with the stem-loop may suggest a new approach to selective tetraplex stabilization.

Our results, described herein, reveal a significant increase in the stability of two QDH structures following the binding of netropsin or Hoechst 33258 to the AATT/AATT sequence of the stem-loop. It follows that, at least for the QDH structures studied in this work, there is a strong thermodynamic coupling between the duplex and tetraplex domains; the coupling allows the binding of a drug to the stem-loop to stabilize the entire structure. Taken together, our results point to a novel, allosteric mechanism for selective stabilization of four-stranded DNA structures with associated structural domains.

## 2. Materials and Methods

### 2.1. Materials

The DNA oligonucleotides d(T_2_G_3_TG_3_T_3_CA_2_T_2_G_2_CACA_2_T_2_GT_3_G_3_TG_3_T) (G4HP), d(CA_2_T_2_G_2_CACA_2_T_2_G) (HP), d(T_2_G_3_TG_3_T_3_CGCGA_2_GCAT_2_CGCGT_3_G_3_TG_3_T) (K6bp6T), d(T_2_G_3_TG_3_TG_3_TG_3_T) (G4), and d(T_2_G_3_TG_3_T_2_CA_2_T_2_GTGCATCA_2_T_2_GT_2_G_3_TG_3_T) (QDH5L) were purchased from Integrated DNA Technologies (Coralville, IA, USA). DNA samples were dissolved in 50 mM CsCl, dialyzed exhaustively against distilled water in Spectra/Por (2000-Da cut-off, Repligen, Waltham, MA, USA), and lyophilized. The lyophilized DNA samples, with the exception of G4, were dissolved in a pH 6.7 buffer, consisting of 10 mM cacodylic acid/cesium cacodylate, 5 mM KCl, and 0.1 mM EDTA. In the presence of 5 mM KCl, G4 and its complexes with drugs are too stable and melt at temperatures which are experimentally unattainable. Therefore, G4 was dissolved in a pH 6.7 buffer, consisting of 10 mM cacodylic acid/cesium cacodylate, 0.2 mM KCl, and 0.1 mM EDTA. Before measurements, all DNA samples were annealed to ensure that, at room temperature, they exist not in a kinetically trapped but in the thermodynamically stable structural state.

Hoechst 33258·3HCl and netropsin·2HCl were obtained from Sigma-Aldrich Canada (Oakville, ON, Canada). The DNA and drug concentrations were determined spectrophotometrically with a Cary 300 Bio spectrophotometer (Varian Canada, Inc., Mississauga, ON, Canada). To determine the concentrations of G4HP, HP, K6bp6T, G4, and QDH5L, we used molar extinction coefficients ε_260_ of 360,200, 146,400, 354,000, 172,800, and 361,800 M^−1^cm^−1^, respectively. These values were computed for the unfolded states at 25 °C from the nearest-neighbor procedure described by Owczarzy [29]. The extinction coefficients of netropsin and Hoechst 33258 are ε_296_ = 21,500 M^−1^cm^−1^ and ε_338_ = 42,000 M^−1^cm^−1^, respectively [30,31]. In CD spectropolarimetric measurements, the concentrations of the DNA were ~30 μM, while, in fluorometric measurements, they were ~50 nM.

### 2.2. Optical Spectroscopy

CD spectral measurements were conducted in a 1-mm path-length cuvette using a JASCO J-1100 Circular Dichroism Spectrophotometer (JASCO, Easton, MD, USA). Fluorescence intensity in DNA samples in the absence and presence of Hoechst 33258 were measured using an Aviv model ATF 105 spectrofluorometer (Aviv Associates, Lakewood, NJ, USA) with a bandwidth adjusted to 2 nm. CD and fluorescence titration profiles were measured at 25 °C by incremental addition of aliquots of a drug solution to the initial amount of DNA solution. The initial volume of DNA was delivered to the cuvette using a 1-mL Hamilton syringe, while aliquots of the drug were added using a 10-μL Hamilton syringe (Hamilton Co., Reno, NV, USA). The syringes were equipped with a Chaney adapter with a relative delivery accuracy of ±0.1%. For fluorescence measurements, the samples were excited at 359 nm, and the intensity of emission light was recorded through a monochromator at 470 nm. In the CD melting experiments, the temperature was changed at a rate of 1 °C per minute. The transitions temperatures, T_M_, were evaluated from analysis of experimental melting profiles using standard procedures for a two-state helix-to-coil transition [32,33,34].

## 3. Results

### 3.1. DNA Structures

**Figure 1:**
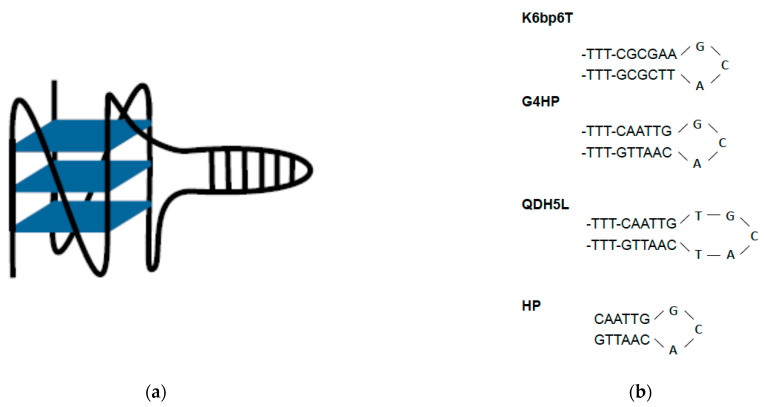
(**a**) Schematic representation of the QDH structures studied in this work; (**b**) the stem-loops of K6bb6T, G4HP, and QDH5L and the hairpin HP.

The QDH-forming DNA sequences studied in this work were the NMR-characterized sequence d(T_2_G_3_TG_3_T_3_CGCGA_2_GCAT_2_CGCGT_3_G_3_TG_3_T), referred to as K6bp6T, and its two derivatives, G4HP [d(T_2_G_3_TG_3_T_3_CA_2_T_2_G_2_CACA_2_T_2_GT_3_G_3_TG_3_T)] and QDH5L [d(T_2_G_3_TG_3_T_2_CA_2_T_2_GTGCATCA_2_T_2_GT_2_G_3_TG_3_T)]. K6bp6T has been shown to form a QHD with a parallel G-quadruplex core and an orthogonally oriented duplex stem [21,22]. This structure is presented schematically in Figure 1a. In G4HP, the six base pairs in the stem-loop of the original construct (CGCGAA/TTCGCG) are replaced with (CAATTG/CAATTG) that contains the (AATT/AATT) binding site for the AT-selective minor groove-binding drugs netropsin and Hoechst 33258. QDH5L contains an AT-containing stem-loop, which is compositionally similar to that of G4HP. However, QDH5L contains a hairpin with a five-nucleotide loop (-TGCAT-) in contrast to the three-nucleotide hairpin loop of G4HP (-GCA-); the stem-loop in the former is more relaxed and should be more B-like than that of the latter. Therefore, one could expect a stronger binding of netropsin or Hoechst 33258 to QDH5L relative to G4HP. The loop sequences of K6bp6T, G4HP, and QDH5L and the sequence of the hairpin (HP) are aligned for comparison in Figure 1b.

**Figure 2:**
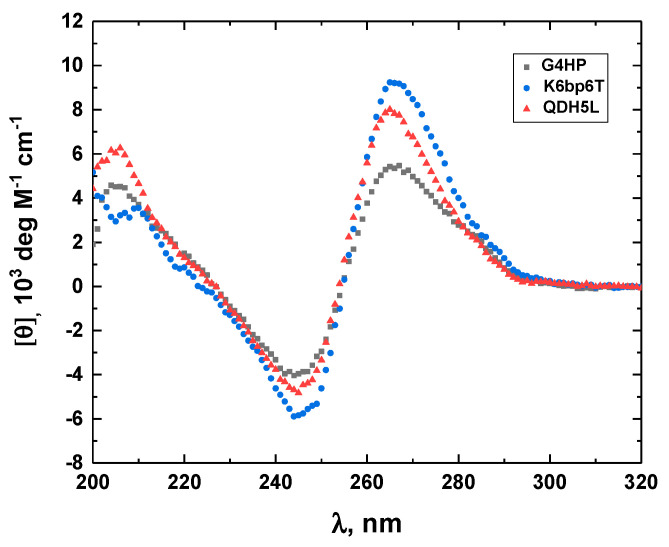
The CD spectra of the G4HP (black), QDH5L (red), and K6bp6T (blue) G-quadruplex–duplex hybrid structures.

To ensure that the mutations introduced to K6bp6T do not change the topology of the G-quadruplex core or the orientation of the stem-loop in the modified QDH structures (G4HP and QDH5L), we compared the CD spectra of G4HP and QDH5L with the spectrum of K6bp6T [22]. Figure 2 shows the three CD spectra; the similarity of the overall shapes of the spectra testifies to the fact that the mutations did not alter the topology of the QDH structures. The differences in the amplitude of the CD bands between G4HP, QDH5L, and K6bp6T may be related to the differential nearest neighbor interactions between the bases of the stem loops.

**Figure 3:**
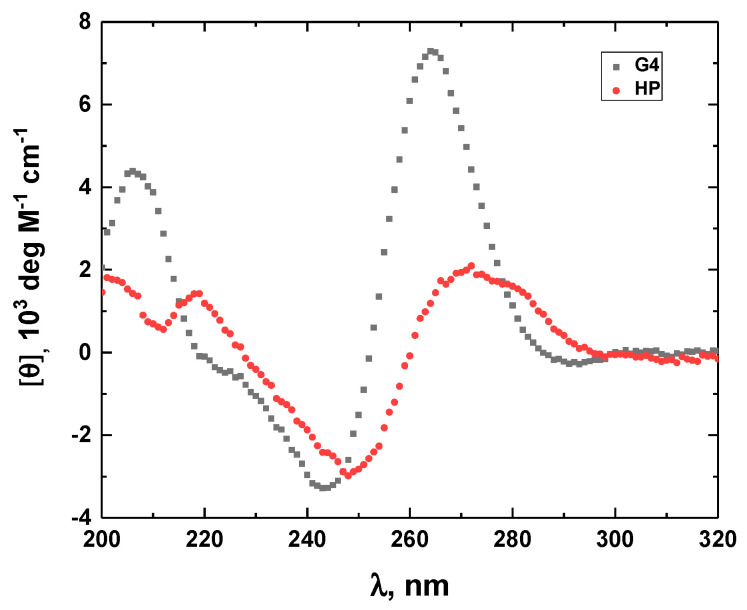
The CD spectra of the G4 G-quadruplex (black) and the HP hairpin (red).

The G-quadruplex G4 with a sequence of d(T_2_G_3_TG_3_TG_3_TG_3_T) and the hairpin HP with a sequence of d(CA_2_T_2_G_2_CACA_2_T_2_G) are the constituent parts of G4HP. We measured the CD spectra of these DNA structures to confirm that, under the experimental conditions of our study, G4 indeed forms a G-quadruplex, while HP folds into a hairpin. The monomolecular nature of the hairpin was additionally verified by UV melting at 260 nm, which revealed no dependence of the melting temperature, T_M_, on DNA concentration. These CD spectra are shown in Figure 3. The CD spectrum of G4 with a negative minimum at 244 nm and a positive maximum at 264 nm is consistent with that of a parallel G-quadruplex.

### 3.2. Drug Binding

**Figure 4:**
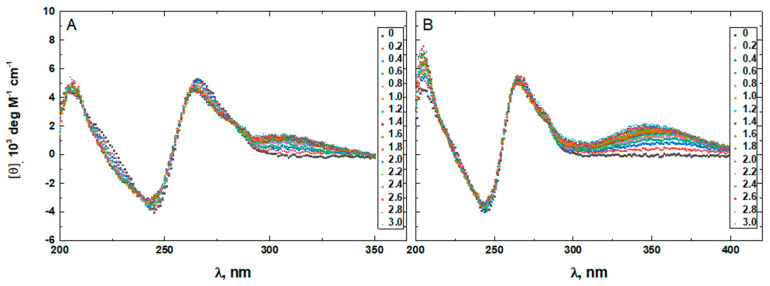
The CD spectra of G4HP in the absence and presence of netropsin (**A**) and Hoechst 33258 (**B**) at various drug-to-DNA ratios, r (shown in the insets), at 25 °C. The DNA concentration is ~20 μM.

**Figure 5:**
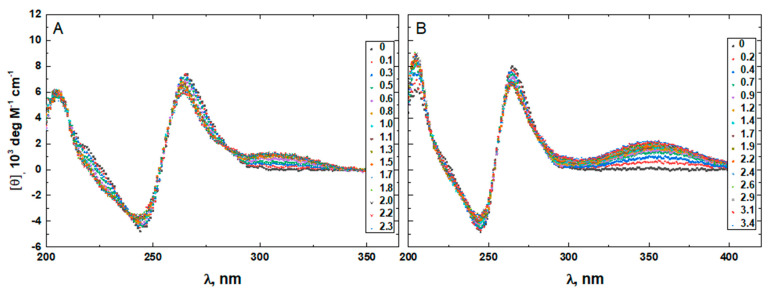
The CD spectra of QDH5L in the absence and presence of netropsin (**A**) and Hoechst 33258 (**B**) at various drug-to-DNA ratios, r (shown in the insets), at 25 °C. The DNA concentration is ~20 μM.

**Figure 6:**
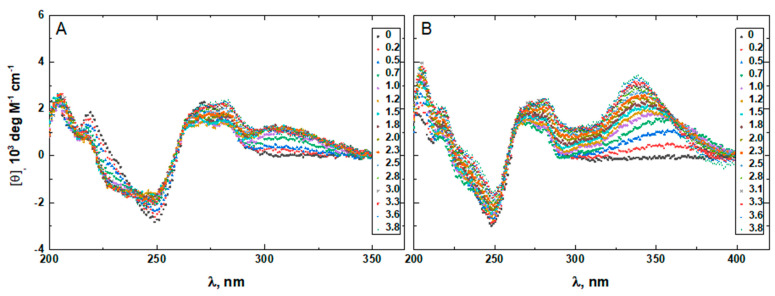
The CD spectra of HP in the absence and presence of netropsin (**A**) and Hoechst 33258 (**B**) at various drug-to-DNA ratios, r (shown in the insets), at 25 °C. The DNA concentration is ~20 μM.

**Figure 7:**
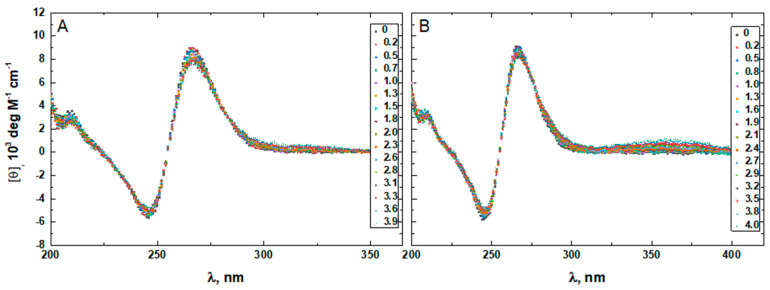
The CD spectra of K6bp6T in the absence and presence of netropsin (**A**) and Hoechst 33258 (**B**) at various drug-to-DNA ratios, r (shown in the insets), at 25 °C. The DNA concentration is ~20 μM.

**Figure 8:**
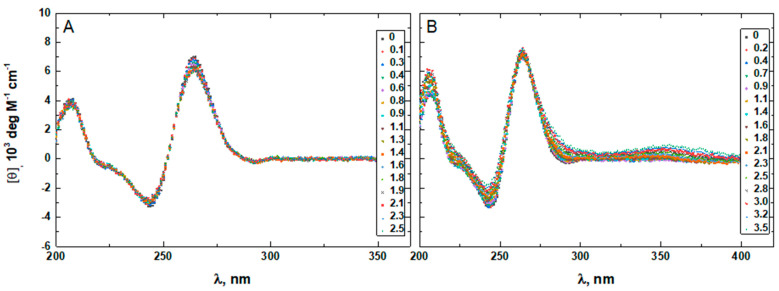
The CD spectra of G4 in the absence and presence of netropsin (**A**) and Hoechst 33258 (**B**) at various drug-to-DNA ratios, r (shown in the insets), at 25 °C. The DNA concentration is ~20 μM.

To confirm that the two minor groove-binding drugs bind to the host structures under the experimental conditions of this study, we measured the CD spectra of each DNA in the absence and presence of netropsin and Hoechst 33258. Additionally, the binding of Hoechst 33258 to the DNA was monitored by fluorescence. The drug-induced CD spectral variations of G4HP, QDH5L, HP, K6bp6T, and G4 are shown in Figure 4, Figure 5, Figure 6, Figure 7 and Figure 8, respectively. In each figure, panel A shows the data on netropsin, while panel B shows the data on Hoechst 33258.

The CD spectra of G4HP, QDH5L, and HP depicted in Figure 4, Figure 5 and Figure 6 exhibit characteristic binding-induced bands at ~315 (panel A) and ~350 (panel B) nm, which are the CD spectroscopic signatures of the association of duplex DNA with netropsin and Hoechst 33258, respectively [35,36]. Thus, both netropsin and Hoechst 33258 bind to the all-AT binding sites of the stem-loops of the G4HP and QDH5L G-quadruplex–duplex hybrids and the hairpin HP. In contrast, the CD spectra of K6bp6T and G4, shown in Figure 7 and Figure 8, either lack or exhibit very weak CD bands above 300 nm. This observation suggests that the two minor groove binders do not bind, or bind only weakly, to the minor groove of a duplex; this is an expected result, given that the G-quadruplex (G4) lacks the duplex portion, while the stem-loop of K6bp6T does not contain the cognate all-AT binding site.

**Figure 9:**
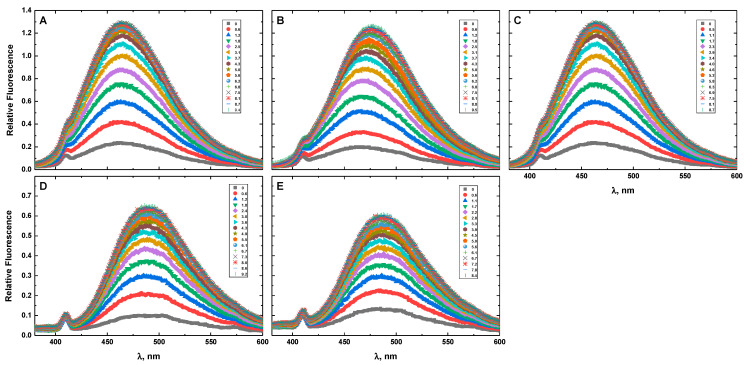
The emission spectra of G4HP (**A**), QDH5L (**B**), HP (**C**), K6bp6T (**D**), and G4 (**E**) in the absence and presence of Hoechst 33258 at various drug-to-DNA ratios, r (shown in the inset), at 25 °C. The excitation wavelength is 359 nm. The DNA concentration is ~50 nM.

Figure 9 presents the fluorescence spectra of G4HP (panel A), QDH5L (panel B), HP (panel C), K6bp6T (panel D), and G4 (panel E) in the absence and presence of Hoechst 33258. The drug-dependent spectral variations shown in Figure 9A–E suggest strong binding of Hoechst 33258 to all DNA structures studied here. This result agrees with the results of previous studies that reported the association of minor groove-binding drugs with G-quadruplex structures [37,38,39,40,41,42].

**Figure 10:**
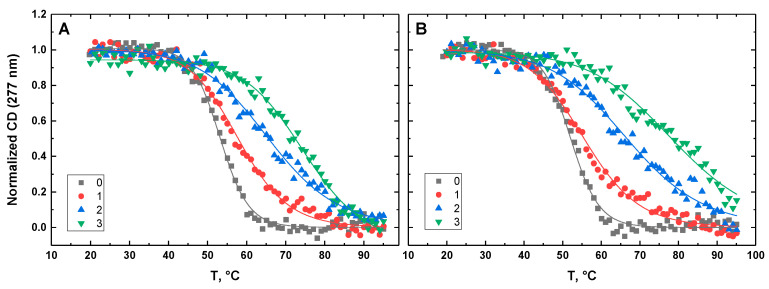
Normalized CD-melting profiles at 277 nm of G4HP in the absence and presence of netropsin (**A**) and Hoechst 33258 (**B**) at various drug-to-DNA ratios, r (shown in the insets). The DNA concentration is ~30 μM. Experimental points were fitted analytically based on the two-state transition thermodynamic formalism [32] (solid lines).

**Figure 11:**
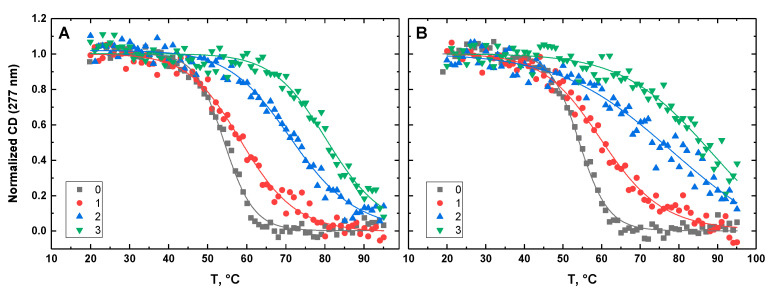
Normalized CD-melting profiles at 277 nm of QDH5L in the absence and presence of netropsin (**A**) and Hoechst 33258 (**B**) at various drug-to-DNA ratios, r (shown in the insets). The DNA concentration is ~30 μM. Experimental points were fitted analytically based on the two-state transition thermodynamic formalism [32] (solid lines).

**Figure 12:**
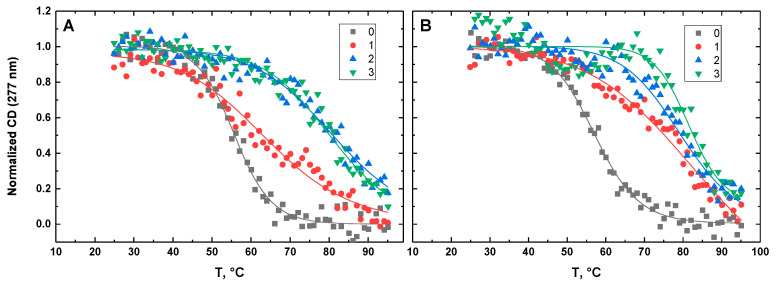
Normalized CD-melting profiles at 277 nm of HP in the absence and presence of netropsin (**A**) and Hoechst 33258 (**B**) at various drug-to-DNA ratios, r (shown in the insets). The DNA concentration is ~30 μM. Experimental points were fitted analytically based on the two-state transition thermodynamic formalism [32] (solid lines).

**Figure 13:**
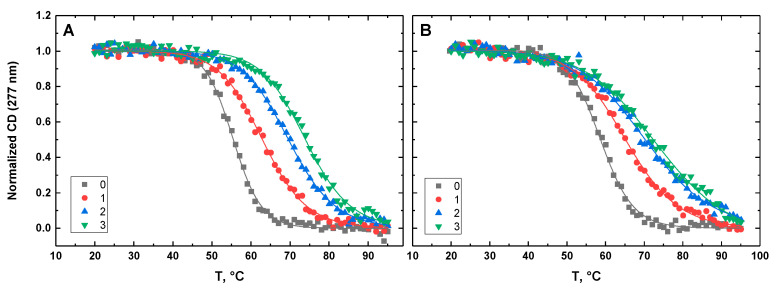
Normalized CD-melting profiles at 277 nm of K6bp6T in the absence and presence of netropsin (**A**) and Hoechst 33258 (**B**) at various drug-to-DNA ratios, r (shown in the insets). The DNA concentration is ~30 μM. Experimental points were fitted analytically based on the two-state transition thermodynamic formalism [32] (solid lines).

Drug-induced spectral changes, such as those shown in Figure 4, Figure 5, Figure 6, Figure 7, Figure 8, Figure 9, Figure 10, Figure 11, Figure 12 and Figure 13, can be used to construct CD- or fluorescence-detected binding profiles at specific wavelengths. Those profiles can then be fitted within the framework of an appropriate binding model, to determine the number of drug-binding sites and their respective affinities. Any such model must accommodate the mutual depletion of DNA and either ligand upon formation of the complex. In case of Hoechst 33258, the model must also accommodate its propensity to aggregate [43]. The effects of mutual depletion and aggregation should be greater at the concentrations of DNA required for measurements of CD (~20 μM) than at those required for measurements of fluorescence (~50 nM). Studies along these lines are in progress. In the present paper, the spectral changes depicted in Figure 4, Figure 5, Figure 6, Figure 7, Figure 8 and Figure 9 are treated as a manifestation of ligand binding rather than a source of quantitative thermodynamic information. We concentrate instead on the binding-induced increases in melting temperatures, ∆T_M_, which represent a global measure of drug-induced stabilization of DNA structures.

### 3.3. Thermal Stability

**Figure 14:**
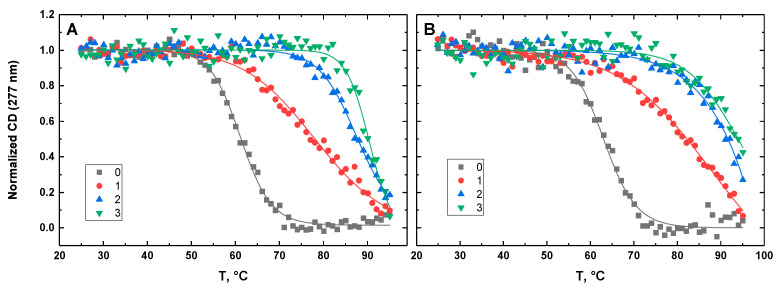
Normalized CD-melting profiles at 277 nm of G4 in the absence and presence of netropsin (**A**) and Hoechst 33258 (**B**) at various drug-to-DNA ratios, r (shown in the insets). The DNA concentration is ~30 μM. Experimental points were fitted analytically based on the two-state transition thermodynamic formalism [32] (solid lines).

Figure 10, Figure 11, Figure 12, Figure 13 and Figure 14 show the CD-detected melting profiles of G4HP, QDH5L, HP, K6bp6T, and G4, respectively, at drug-to-DNA ratios from 0 to 3. In each figure, panel A presents melting profiles in the presence of netropsin, while panel B shows the thermal data in the presence of Hoechst 33258. Inspection of Figure 10, Figure 11, Figure 12, Figure 13 and Figure 14 reveals that the binding of either drug to each DNA structure studied here results in its stabilization, as can be judged by significant increases in T_M_ at increasing drug-to-DNA ratios.

Below, we derive the equation for the ligand-induced increase in T_M_ for a biopolymer with multiple ligand-binding sites. We assume that the biopolymer may exist in either the native, *N*, or denatured, *D*, state and that the ligand binds only to the native state. The equilibrium between the native state with n ligand-binding sites and the denatured state (which does not bind ligands) is given by the relationship:

(1) $$K=\frac{\left[D\right]}{\left[N\right]}=\frac{\left[D\right]}{[N_{0}]\prod_{i=1}^{n}\left(1+k_{i}\left[L\right]\right)}=\frac{K_{0}}{\prod_{i=1}^{n}\left(1+k_{i}\left[L\right]\right)}$$

where $K_{0}=\frac{\left[D\right]}{\left[N_{0}\right]}$ is the equilibrium constant between the native and denatured states of the biopolymer in the absence of the ligand; *k_i_* is the microscopic affinity of the ligand for the *i*-th binding site; [*L*] is the concentration of free ligand; and $\overset{n}{\underset{i=1}{\prod }}(1+k_{i}\left[L\right])$ is the binding polynomial [44].

The stability of the ligand–biopolymer complex is given by the equation:

(2) $$\Delta G=-\mathrm{RTlnK}=\Delta G_{0}-\Delta G_{b}=\Delta H_{0}-T\Delta S_{0}+\mathrm{RTln}[\prod_{i=1}^{n}(1+k_{i}\left[L\right])]$$

where ∆G_0_ = −RTlnK_0_ = ∆H_0_ − T∆S_0_ is the stability of unligated biopolymer; ∆H_0_ and ∆S_0_ are the respective changes in enthalpy and entropy; ∆G_b_ = −RT$\overset{n}{\underset{i=1}{\prod }}(1+k_{i}\left[L\right])$ is the binding free energy.

At the melting temperature, T_M_, the value of ∆G = 0. Thus, one obtains the following relationship by equating Equation (2) to zero:

(3) $$\Delta H_{0}-T_{M}\Delta S_{0}+{\mathrm{RT}}_{M}\ln[\prod_{i=1}^{n}(1+k_{i}\left[L\right])]=0$$

From Equation (3), T_M_ is given by the relationship:

(4) $$T_{M}=\Delta H_{0}/(\Delta S_{0}-\mathrm{Rln}[\prod_{i=1}^{n}(1+k_{i}\left[L\right])])$$

By dividing the numerator and the dominator of RHS of Equation (4) by ∆H_0_ and taking into account that the melting temperature of unligated biopolymer, T_M0_, equals ∆H_0_/∆S_0_, one obtains the following:

(5) $$\Delta T_{M}=T_{M}-TM_{0}=(\mathrm{RT}M_{0}T_{M}/\Delta H_{0})\;\ln[\prod_{i=1}^{n}(1+k_{i}\left[L\right])]$$

where T_M0_ and T_M_ are the melting temperatures of the biopolymer at free ligand concentrations of 0 and [*L*], respectively.

For *n* = 1, Equation (5) converts into the well-known equation derived by Crothers in 1971 [25]. To the best of our knowledge, Equation (5), relating the melting temperature of a biopolymer with multiple binding sites to the concentration of a ligand and its affinity for the sites, has not been reported previously. Inspection of Equation (5) reveals that ∆T_M_ represents an unambiguous global measure of the net drug-induced stabilization of DNA structures.

We fitted the melting profiles presented in Figure 10, Figure 11, Figure 12, Figure 13 and Figure 14 by a two-state transition model [32]. The values of T_M_ were determined from the fits as the helix-to-coil transition midpoints. Table 1 presents increases in T_M_ for each DNA construct studied here in the presence of netropsin and Hoechst 33258 at a drug-to-DNA ratio, r, of 3.

## 4. Discussion

The minor-groove binding drugs studied here associate with the G-quadruplex domain of a G-quadruplex–duplex hybrid structure and its stem-loop if the latter contains the all-AT binding site. As alluded to above, a change in T_M_ represents a quantitative measure of the cumulative effect of drug binding on the structural stability of DNA. Inspection of the data on T_M_ presented in Table 1 reveals a hierarchical order of drug-induced stabilization, which is consistent with the following picture. Both netropsin and Hoechst 33258 bind to the G-quadruplex G4 and strongly stabilize it, as suggested by increases in T_M_ of 29.3 ± 0.5 and 31.8 ± 3.8 °C, respectively. It follows, by extension, that the two minor groove-binding drugs also bind to the G-quadruplex domain of the G4HP, QDH5L, and K6bp6T hybrids. Comparison of the stability data on K6bp6T and G4 reveals that the presence of the stem-loop in the hybrid causes a large reduction in the extent of drug-induced stabilization; the values of ∆T_M_ diminish to 18.5 ± 0.2 and 14.8 ± 0.4 °C for netropsin and Hoechst 33258, respectively. The stem-loop in K6bp6T lacks the all-AT binding site and, therefore, cannot associate with either of the two drugs. The observed decrease in the extent of drug-induced stabilization of K6bp6T suggests that the stem-loop either abolishes or sterically hinders the access of the drugs to one or more of the binding sites on its G-quadruplex domain.

In contrast to K6bp6T, the stem-loop of G4HP contains an all-AT binding site. Hence, netropsin and Hoechst 33258 bind, in addition to the G-quadruplex domain, to the stem-loop of G4HP, which is reflected in the CD spectra in Figure 4A,B. Comparison of the drug-induced increases in T_M_ for G4HP and K6bp6T reveals that complexation of the minor groove-binding drugs with the stem-loop of G4HP leads to its stabilization, which significantly exceeds that afforded by the drug binding to K6bp6T. The values of ∆T_M_ for G4HP are 21.5 ± 0.4 and 26.4 ± 0.3 °C for netropsin and Hoechst 33258, respectively. Coincidentally, these numbers are close to those observed for the HP hairpin, where the values of ∆T_M_ are 24.7 ± 0.4 and 24.4 ± 0.8 °C for netropsin and Hoechst 33258, respectively.

These results are consistent with the notion that the drug-induced stabilization of G4HP reflects the concerted effect of drug binding to both the stem-loop and the G-quadruplex domain of the hybrid. Comparison of the stability data for QDH5L and G4HP provides further evidence that drug binding to the stem-loop contributes significantly to the stabilization of a hybrid structure. QDH5L contains a five-loop hairpin in contrast to the more constrained three-loop hairpin of G4HP. The unconstrained stem-loop duplex in QDH5L should be more B-like relative to G4HP; hence, the binding of the minor groove binders to the stem-loop of QDH5L should be tighter than that to the stem-loop of G4HP. One can, therefore, expect stronger drug-induced stabilization of QDH5L than of G4HP. In agreement with this expectation, the values of ∆T_M_ for QDH5L were 26.6 ± 0.7 and 35.9 ± 0.4 °C for netropsin and Hoechst 33258, respectively, significantly exceeding those for G4HP. Therefore, an increase in the number of the nucleotides in the loop of the hairpin from three to five leads to substantially enhanced drug-induced stabilization of the QDH structure.

Our results suggest that minor groove-binding drugs stabilize QDH structures by binding to both the stem-loop and the G-quadruplex domains. While the contribution of drug binding to the G-quadruplex domain is large, the contribution of the binding to the stem-loop is also substantial. The two contributions are difficult to separate, and the combined effect of drug binding to the stem-loop and the G-quadruplex domain may be synergistic.

Our results may seem to contradict those of a study that suggested that netropsin binding to the hairpin stem-loop portion of one particular QDH does not lead to any appreciable increase in stability, despite enhancing the stability of the isolated hairpin [24]. The observed disparity reflects the structural differences in the QDH structures studied in this work and that studied by Nguyen et al. [24]. In the QDH studied in ref. [24], a nick is introduced to the G-tract bordering the quadruplex–duplex junction. Thus, in that structure, the stem-loop hangs from the 3′-end of the tetraplex core and is anchored to its terminal G-tetrad, which serves as the foothold for the loose end of the hairpin. The latter is not a loop in the tetraplex core of the hybrid structure, in the sense that it does not link G-runs; it is rather an appendix which is structurally and thermodynamically separate from the core.

The data described in the present work outline an untapped avenue for sequence-selective recognition and stabilization of stem-loop-containing QDH structures in vitro and, possibly and more importantly, in vivo. Given the existing library of minor groove-binding drugs recognizing mixed AT- and GC-rich DNA sequences [45,46,47,48,49], our results outline a potential method for stabilization, or even induction, of QDH structures with stem-loops containing cognate sequences, with clear extensions to, and implications for, the control of genomic events.

There is a subtle point that merits discussion. The thermodynamic data presented in this work leave no doubt that drug binding to the stem-loop contributes significantly to an increase in the stability of an isolated QDH. It is less clear if the same or a similar extent of drug-induced stabilization would be afforded within the context of duplex DNA, when the QDH is exposed to its complementary C-rich strand. In this scenario, the QDH-plus-single strand conformation with a single drug-binding site should compete with the duplex conformation with two drug-binding sites (given the palindromic nature of the stem-loop). The equilibrium between the QDH and duplex species would, clearly, depend on the specific DNA sequence and the identity of the drug; the QDH conformation may or may not outcompete the duplex conformation. Further studies are needed to explore the effect of drug binding on duplex–QDH equilibrium.

## 5. Conclusions

We characterized the effect of the minor groove binders netropsin and Hoechst 33258 on the stability of a family of QDH structures, as well as a G-quadruplex and a hairpin modeling the G-quadruplex core and stem-loop of the QDH’s. The binding of either drug results in an increase in thermal stability for all DNA structures, as evidenced by increases in melting temperature, T_M_. Analysis of the hierarchical order of increases in T_M_ reveals that the drug-induced stabilization arises from drug binding to both the G-quadruplex domain of a QDH and its stem-loop if the latter contains an all-AT binding site. This result attests to the thermodynamic crosstalk between the stem-loop and tetraplex core of the QDH. Our results allude to an untapped avenue for sequence-specific recognition of QDH structures.

## Figures and Tables

**Table 1:** Ligand-induced changes in melting temperatures, ΔT_M_ (°C), of DNA structures at a drug-to-DNA ratio, r, equal to 3.

	G4HP	HP	K6bp6T	G4	QDH5L
ΔT_M_ (Netropsin)	21.5 ± 0.4	24.7 ± 0.4	18.5 ± 0.2	29.3 ± 0.5	26.6 ± 0.7
ΔT_M_ (Hoechst 33258)	26.4 ± 0.3	24.4 ± 0.8	14.8 ± 0.4	31.8 ± 3.8	35.9 ± 0.4

## Data Availability

Not applicable.
